# Considering Farmers’ Heterogeneity to Payment Ecosystem Services Participation: A Choice Experiment and Agent-Based Model Analysis in Xin’an River Basin, China

**DOI:** 10.3390/ijerph19127190

**Published:** 2022-06-11

**Authors:** Shengnan Li, Baohang Hui, Cai Jin, Xuehan Liu, Fan Xu, Chong Su, Tan Li

**Affiliations:** 1 College of Economics and Management, Anhui Agricultural University, Hefei 230036, China; lishengnan19970406@163.com (S.L.); hbh@stu.ahau.edu.cn (B.H.); jinc612@outlook.com (C.J.); l18053796938@163.com (X.L.); 2 College of Economics and Management, South China Agricultural University, Guangzhou 510642, China; xf0312@stu.scau.edu.cn; 3 Institute of Agriculture Remote Sensing and Information Technology, College of Environmental and Resource Sciences, Zhejiang University, Hangzhou 310058, China; suchong@zju.edu.cn

**Keywords:** ecological compensation, choice experiment, agent-based model, China, Xin’an River Basin

## Abstract

The concept of watershed ecological compensation is one payment for ecosystem services (PES) program that incentivizes stakeholders undertake environmental conservation activities that improve the provision of ecosystem services. Defining the heterogeneity of farmers’ willingness to participate in watershed ecological compensation is critically important for fully understanding stakeholders’ demands. Accordingly, we designed a choice experiment survey to analyze the heterogeneity of policy preferences and willingness to receive compensation between upstream and midstream farmers in Xin’an River basin, China. Moreover, we simulated the impact of farmers’ social capitals’ heterogeneity with an agent-based model. The results show that there are significant differences in the preferences of agricultural waste recycling rate and agricultural water quality between farmers in the upstream and midstream. The total willingness of farmers in the upstream and midstream to participate in ecological compensation are RMB 149.88 (USD 22.54)/month and RMB 57.40 yuan (USD 8.63)/month, respectively. Social network size has a negative effect on farmers’ willingness to participate the programs. Our findings suggest that the characteristics of farmers’ influence their willingness to participate in the PES program. The results of this research can be used to improve PES management policies in the future, as well as to support sustainable environmental development and rural revitalization.

## 1. Introduction

Payments for ecosystem services (PES) is an effective policy tool to balance the coordinated development of socioeconomic activities and ecological protection [1,2,3], especially in developing countries. Currently, PES has been used in some environmental management programs widely, such as forest [4,5,6,7,8], cropland [9,10,11,12], and watershed [13,14,15]. This growing trend is evident in China as well.

The value of ecosystem services has been extensively studied since Costanza’s assessment in 1997 [16], when scholars conducted extensive research on global [17], regional [18], and locally specific ecosystem service values [19]. As the PES policy is primarily adopted in a small number of developed and developing countries, it has not been promoted globally. On the other hand, an analysis of participation in implementing PES policies in typical regions will assist its global implementation. PES programs face several challenges that must be overcome to achieve long-term success. In a study of 40 PES programs in Latin America, only 57% were deemed successful for promoting ecological, economic, or social well-being [20]. A program could be successful if it fulfills the following four criteria: (1) provide a valuable resource while contributing to local livelihoods, (2) operate on a local or regional scale, (3) utilize in-kind contributions in addition to cash payments, and (4) involve private actors and reduce middlemen between buyers and sellers of ES. By designing PES programs based on local marketplaces’ needs and characteristics, previous research argues for taking a non-commodity view of watershed services [21].

In China, ecological compensation is a typical PES program [22,23]. In 2012, the Xin’an River ecological compensation was officially launched, becoming the first cross-regional watershed ecological compensation nationwide. In 2017, the nation proposed to reasonably determine compensation standards and promote the establishment of a long-term mechanism for ecological compensation. Subsequently, the Xin’an River ecological compensation has been taken as a typical program in China [24]. However, the low willingness of farmers to participate in the policy has become a tough issue in the policy implementation process.

Although the logic of PES schemes seems uncomplicated, paying farmers in exchange for a service is by no means a straightforward task [25]. Ecosystem services result from a number of ecological interactions [26]. These interactions are underestimated by science. There is no guarantee that a payment will provide a service; this applies not only to underlying ecological uncertainties but also to those enrolled by the social interaction and the farmers’ characteristics to policy implementation [27].

Previous studies have estimated the factors influencing farmers’ willingness to participate in PES policies in terms of their own characteristics, such as household characteristics [28,29,30,31] and social capitals [32,33,34]. Olson proposed the theory of collective action based on the “rational economic person” hypothesis [35]. In Olson’s view, rational, self-interested individuals will unite to achieve common interests, provided that the group is small. Because small groups allow for avoiding the free-rider problem. Olson’s free-rider analysis applies to large groups. If the group is sufficiently small, each individual’s contribution to the collective goods will make a difference. Each person will contribute as long as their benefits in the common good outweigh the costs. In the ecological compensation of Xin’an River, ecological environment means a public good, which is non-competitive and non-exclusive. If farmers living nearby the river basin find that their costs outweigh the benefits, it will reduce their willingness to participate in the policy and leads to the dilemma of collective action.

Meanwhile, equity implications and asymmetric power distribution between actor groups should be paid more attention. Some scholars claim that the power imbalances and the inequalities might be reinforced by the design of PES [36]. Farmers, as important stakeholders of the policy [22], are not only protectors of the environment but also implementers of the policy. In a watershed PES in Xin’an River in China, the scheme is designed as the downstream compensate the protectors from the upstream and middle stream. However, the farmers’ enthusiasm of participation in PES are different between upstream and middle stream. This phenomenon may be related to their preferences to PES.

Choice experiments (CE) is an effective tool for analyzing farmers’ preferences, and many scholars have applied choice experiments to areas related to ecological compensation [37,38,39,40,41]. The CE method provides estimation to more information and enables simultaneous analysis of farmers’ preferences for policy packages [42]. This allows for greater flexibility and better comparison of different compensation policies [43]. In this paper, we implemented CE for farmers upstream and downstream of the Xin’an River and analyzed the data using a Random Parameter Logit (RPL) model to explore farmers’ preferences among different ecological compensation policy schemes. Based on the RPL model, we quantified the willingness to participate and calculated the amount that farmers expect to receive during the policy implementation period. Nevertheless, the land use and the potential effects of agricultural and forestry resources management may be different between upstream and middle stream. Thus, the farmers from these two areas are heterogeneous in collective actions.

Collective action involves complicated subjects of interest, and its results emerge from the interaction and decision making of multiple subjects [44,45]. These interactions between farmers’ economic activities and their land use decision are even more critically challenging in mountainous areas with command-and-control or indirect conservation incentives [46]. Despite the recognized complexity in the dynamics of the rural land system and farmers’ participation in PES [47], how the rule of local payments ecosystem services affects farmers’ preferences requires further investigation, which is difficult to observe in econometric models.

The agent-based model (ABM) can simulate this process in the framework of coupled natural and human systems [48]. One advantage of ABM is that it is a “bottom-up” approach that can directly represent the interaction of individual agents’ decision-making processes with ecological compensation policies and the environment [49,50]. ABM is widely used by researchers in social–ecological systems to solve complex problems [51,52,53,54]. In this paper, we use ABM to simulate farmers’ willingness to participate in current policy scenarios versus policy contexts that incorporate preferences, and our findings provide support for addressing the collective action dilemma.

The rest of this paper is organized as follows: Section 2 introduces methodology including the research area, the sampling and the models used for analysis. Section 3 shows descriptive and model results. Section 4 discusses the results and policy implications.

## 2. Methods

### 2.1. The Study Areas and Sampling

**Figure 1:**
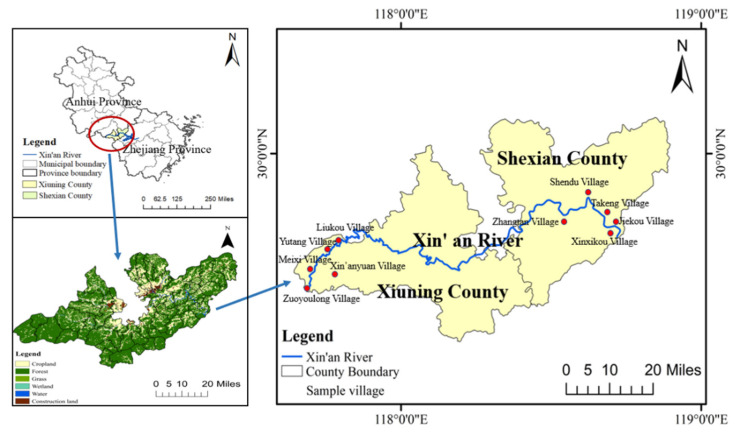
Location of Xin’an River.

Xin’an River originates from Xiuning County in Huangshan City, Anhui Province, China (117°38′–119°21′ E, 29°11′–30°20′ N) (Figure 1). This river stretches over 373 km with a total catchment area of approximately 11,000 km^2^ [55]. It is a strategic water source and an important ecological barrier for the integrated development of the Yangtze River Delta.

Since the implementation of the ecological compensation policy in 2012, the ecological environment of the Xin’an River basin has been well improved. To reach an agreement between Anhui Province, which is located in the upstream and midstream, and Zhejiang Province, which is located in the downstream, to cooperate in the management of the Xin’an River, a national water quality monitoring station was built in She County to monitor water quality monthly. To protect the source water quality, upstream and middle stream farmers are prohibited from using pesticides and fertilizers and are very strict about the recycling of agricultural waste. As a result, the local government is very strict about water quality and has banned all fishing activities by farmers. In the Xin’an River basin or other areas in developing countries, farmers often use some polluting method to fish, such as bait and pesticide, which will pollute the water quality.

Since 80% of the Xin’an River basin is in the upper and middle reaches, the upper and middle reaches of the basin have to undertake more environmental governance tasks and impose higher restrictions on the production and living of rural residents to ensure water quality and quantity [23]. Xiuning County in Huangshan City is located in the upper stream of the Xin’an River (Figure 1). The county has implemented a centralized distribution of pesticides and fertilizers. Local rural residents have more production and living restrictions than those in the middle and lower reaches, but rural residents in this area receive less direct economic compensation. She County is located in the middle stream of the Xin’an River. In this area, there are many ashore fishermen and immigrants from the reservoir area. The policies that these rural residents can obtain economic subsidies and various forms of transfer employment opportunities.

We anchored the sampling in Xiuning County and She County, Huangshan City, Anhui Province in 2020. The investigation was carried out in two processes. The first stage is pre-research. We first identified a script for an interview and trained the team members. Then, members of the subject group conducted pre-research in August 2020 in Xiuning County and She County. This was beneficial to understand the current status of the implementation of ecological compensation policy in Xin’an River basin, the basic situation of local farmers and their willingness and attitude to participate in ecological compensation policy. Additionally, through discussions with experts in the field of ecological compensation, we determined the attributes of the choice experiment chosen. The second stage is formal investigation. From November to December 2020, we selected farmer household samples through stratified random sampling and carried out a selection experimental investigation. The specific steps are: firstly, randomly select 2 to 3 towns nearest to the Xin’ an River in the sample county; then, randomly select 2 to 3 villages in each town; lastly, randomly select about 20–30 farmers in each village to conduct a questionnaire survey.

A total of 300 questionnaires were distributed, and after eliminating some invalid questionnaires and data, 290 valid questionnaires were obtained, with an effective rate of 96.7%.

### 2.2. Elicitation of Farmers’ Preferences

This paper used the discrete choice experiment method (CE) to elicit farmers’ preference for attributes of ecological compensation programs and the tradeoffs among the attributes to better understand the driving factors of their decisions. CE is a survey-based stated preference method [56]. The method is increasingly applied in policy studies to gain insights on how to better design and deliver practices to meet the needs of small farmers [57,58].

We followed three steps to implement the CE. First, we identified the attributes related to the local PES program. We performed a focus group discussion with local village committees and farmers. The purpose was to understand the local situation to determine the farmers’ concerns about the policy and the impact of the policy on the farmers. After the discussion, we interviewed several experts in the field of ecological economics and, finally, determined five attributes. Table 1 shows the choice of attributes and attribute levels.

The first attribute, “livestock and poultry breeding”, was chosen to describe the possibility of the farmers willingness to breed livestock and poultry. Anhui Provincial Government has implemented the Three-Year Action Plan for Resourceful Utilization of Livestock and Poultry Breeding Waste in Anhui Province (2018–2020) in order to protect the ecological environment of Xin’an River. At present, in order to protect the water resources of Xin’an River and improve the local ecological environment, the ecological compensation policy not only requires the closure of large farms, but also prohibits farmers from raising chickens, pigs and other livestock at home. These administrations have a certain impact on farmers’ lives. Therefore, we set two levels of comprehensive ban and rationalized livestock farming, and set the comprehensive ban as the status quo.

The second attribute “agricultural water quality” refers to the grade of agricultural water quality that framers prefer to. Water quality is a reference for the deal of Anhui and Zhejiang provinces. In order to meet the requirements of the prescribed grade II water quality, upstream and midstream farmers are subject to many restrictions in their production and life. The agricultural water quality may directly affect the quality of crops and farmers’ income. Therefore, we set two levels of grade I water quality and grade II water quality, and the current situation of Xin’an River water quality is grade I water quality.

The third attribute, “agricultural waste recycling rate”, means the recycling rate that the farmers deal with their agricultural waste: resource utilization of agricultural waste is an effective way to improve the ecological environment of Xin’an River and prevent agricultural surface source pollution. In this paper, based on the “Guidance on Fertilizer Packaging Waste Recycling Treatment” issued by the Ministry of Agriculture and Rural Affairs in 2020, four recycling levels of 75%, 80%, 85%, and 90% were set. Additionally, a 85% recycling rate of agricultural waste was set as the status quo.

The fourth attribute, “compensation years”, captures the average period that farmers want to be compensated. Compensation years are the length of time that farmers are willing to participate in ecological compensation policies and expect to receive compensation. At present, Xin’an River ecological compensation has been carried out for three rounds of pilot projects. Knowing the expected number of years of compensation for farmers can help develop reasonable compensation methods and compensate for farmers’ losses. In this paper, the compensation years levels of 3, 5, 7, and 9 years are set, and the status quo is set as 5 years.

The fifth attribute, “cash requirement”, represents the average amount of money that a farmer needs to be compensated or willingness to accept (WTA). In order to cover the cost of farmers’ participation in ecological compensation losses, the government will distribute a certain amount of compensation each month, but the upstream farmers are not compensated with the compensation funds. Data were obtained through a pilot survey, and focus group discussions informed the range of the attribute levels for cash requirements. We set the monthly compensation amount for each farmer as RMB 25 (about USD 3.76), RMB 50 (about USD 7.52), and RMB 75 (about USD 11.28) to measure the willingness of farmers to participate in the policy to be compensated (according to the current exchange rate in June 2022, US 1 dollar = RMB 6.647). According to data provided by the focus group, RMB 75 equals 25% of agricultural income per month for local farmers.

Second, as per the setting of attribute levels shown in Table 1, 2 × 2 × 4 × 4 × 3 = 192 alternatives are obtained. We used the experiment design to combine the various attributes and levels into different pairs of mutually exclusive hypothetical options of choice sets. Therefore, we generated an orthogonal design with JMP software to maximize the design efficacy (D-efficiency), and the D-efficiency was 85.62%, indicating an acceptable precision of orthogonality of the options. We constructed 6 choice sets, and the choice sets were randomly divided into two versions of the questionnaire, each containing 3 choice sets, and each choice set contained 2 hypothetical options and 1 option to maintain the status quo. Moreover, each questionnaire includes a screening choice set to exclude invalid respondents. An example of the choice sets is shown in Table 2.

Finally, we randomly assigned the sample farmers to one of the two choice cards. Before commencing the CE, a detailed explanation was introduced to the farmers. It included the purpose of the CE, the attributes and levels. In the process, we showed each sample six choice cards after the other in a random order, which could avoid ordering effects. We performed a follow-up survey after this process to ask farmers their viewpoint about the CE and the attributes. The survey was implemented in 2020 via a face-to-face interview by trained investigators and supervisors.

### 2.3. Choice Experiment Framework

The CE modeling framework relies on the Lancaster consumer choice model [59] and the random utility theory [60]. Lancaster’s framework proposes that people’s demand for goods does not come from the goods themselves, but the attributes are contained in the goods. Therefore, the value of a commodity is the sum of all its characteristic values. In the choice experiment, it can be expressed as the commodity in terms of a set of attributes. The theory of random utility proposed by McFadden [60] holds that the true utility of consumer consumption of goods is divided into observable definite utility and non-observable random utility. Based on these two theories, we assume that the random utility of farmers’ is composed of the deterministic components and random components under the utility maximization hypothesis, the utility that farmer i obtains from alternative j can be presented as:

(1) $$U_{ij}=V_{ij}+\varepsilon_{ij}$$

where $V_{ij}$ represent observable utility on farmers’ choices, and the random variable $\varepsilon_{ij}$ represents unobservable utility.

Farmer i chooses alternative j on choice card if and only if $V_{ij}>V_{in}$, ∀j≠n. Therefore, the probability that farmer i choose alternative j can be expressed as:

(2) $$P_{ij}=P(V_{ij}+\varepsilon_{ij}>V_{in}+\varepsilon_{in})\forall j\neq n,n\in C$$

The observable utility can be expressed as:

(3) $$V_{ij}=ASC+\sum \beta_{jk}X_{jk}$$

We define the Alternative Specific Constant (ASC) as a dummy variable, which takes the value equals “1” for status quo and “0” otherwise. $X_{jk}$ assumes the k*th* attribute variable in scenario j, and $\beta_{jk}$ is the corresponding coefficient.

The multinomial logit (MNL) assumes homogeneous preferences among individuals [61]. Random parameter logit (RPL) performs better as it captures the heterogeneity of farmers’ preferences [62], so we chose the RPL (see details in Text S1). In the RPL model, to better explain the effect of differences in farmers’ characteristics on the alternative, we added interaction terms of ASC with variables such as gender ($G_{i}$), age ($A_{i}$), education ($E_{i}$), number of household laborers ($NL_{i}$), forestland area ($WA_{i}$), forestland slope ($WS_{i}$), distance from residence to river ($DR_{i}$), and distance from forestland to river ($DW_{i}$) in Formula (4). γ is the coefficient to be estimated for each interaction term. The formula is as follows:

(4) $$\begin{array}{l} V_{ij}=\alpha ASC+\sum \beta_{jk}X_{jk}+\sum \gamma \cdot ASC\cdot G_{i}+\sum \gamma \cdot ASC\cdot A_{i}+\\ \sum \gamma \cdot ASC\cdot E_{i}+\sum \gamma \cdot ASC\cdot NL_{i}+\sum \gamma \cdot ASC\cdot WA_{i}+\\ \sum \gamma \cdot ASC\cdot WS_{i}+\sum \gamma \cdot ASC\cdot DR_{i}+\sum \gamma \cdot ASC\cdot DW_{i}+\varepsilon \end{array}$$

In choice experiments, one of the attributes is used as a monetary measure of the willingness to accept [63]. By estimating each attribute parameter by the maximum likelihood method, the WTA of farmers to participation can be obtained. The formula is as follows:

(5) $$WTA=-\frac{\beta_{attribute}}{\beta_{compensation}}$$

where the $\beta_{attribute}$ is the coefficient of the attributes, and $\beta_{compensation}$ is the coefficient of the monetary compensation attribute.

### 2.4. Agent-Based Model (ABM) Analysis

Although the CE method could clarify the farmers’ heterogeneous preferences and the monetary value of their WTA in participation in PES, interactions among the farmers are difficult to observe. For instance, the farmers’ social networks and their social trust may influence each other and generate iterations. However, these iterations cannot be calculated in econometrical models. The agent-based model (ABM) is a method developed based on the complex adaptive system, which could capture these iterations [54,64]. It simulates each agent from the bottom up by endowing the micro-subject with certain attributes, behavior rules, and interaction mechanisms to study the phenomenon at the macro-level [65,66]. To observe the influences of the social networks and social trust of farmers, we developed the ABM model with the Net logo 6.2.1 software. In this part, we provide an overview of the ABM, while a detailed description of the model following the ODD (overview, design concepts, and details) protocol [67,68,69,70] is given in the Supplementary Materials (Text S1). The model started in 2020, when survey data were collected. Each simulation proceeds in an annual time step and runs for 20 time steps. Thus, the model can be used to simulate and predict the participation of households in different ecological compensation policies during the period 2020–2040.

**Figure 2:**
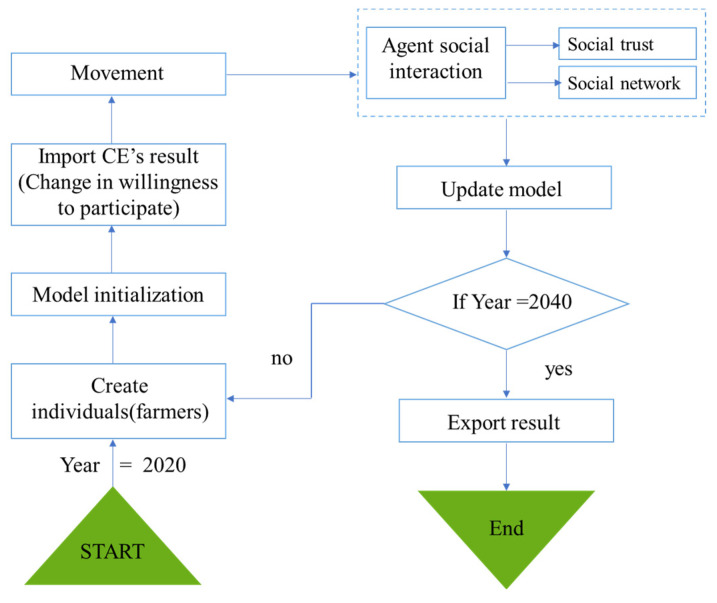
Flow chart of process overview of the agent-based model.

**Figure 3:**
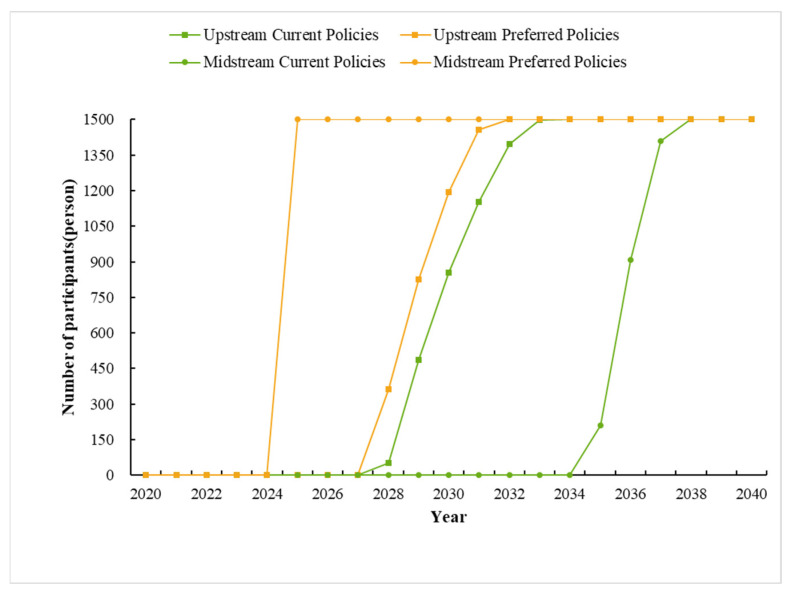
Number of participating farmers in the two policy scenarios.

The flow chart of the ABM is shown in Figure 2. The modeling process can be divided into three stages: initialization, simulation, and output. (1) The initialization stage is to set the initial willingness of farmers to participate in the policy and assign values to each farmer’s gender, age, education, number of laborers, forestland area, forestland slope, distance from residence to river, and distance from forestland to river. These data come from field research. (2) The simulation stage includes three processes: increase or decrease in farmers’ willingness value, movement, and social interaction. The increase or decrease in willingness value depends on the result of CE. In the social interaction part, we determined the parameter values based on the number of farmers with “how many mutual labor relations” in the survey data. We also considered the effect of different levels of social trust degree (0, 25%, 50%, 75%, 100%) (specific results are in Text S1) on farmers’ decision making and, finally, showed the results for the parameter 0.5 (Figure 3). (3) After repeating the above steps for 30 time steps (if year = 2040), ABM enters the output stage.

## 3. Results and Discussion

### 3.1. Descriptive Statistics

We obtained a total of 290 usable samples, 130 from upstream Xiuning County and 160 from midstream She County. Descriptive statistics of the survey sample are shown in Table 3.

Upstream and midstream households may have systematic differences on household characteristics. We compare these characteristics of participators from upstream and midstream (Table 3). The results show that the listed characteristics are significantly difference between these two groups, except gender and forest slope.

The education years of upstream farmers is about 5.48, lower than that of midstream farmers (8.08 years). In terms of gender and age, the proportion of men is generally higher than that of women in both upstream and midstream farmers. The average age is around 61 years old. In terms of household labor, the number of laborers in the upstream was higher than that in the midstream. In terms of forest land area, the upstream farming households owned an average of 1.97 ha, while the forest land area of the midstream farming households was about 0.24 ha. Both upstream and midstream forest lands are steeper. The distance between participators and their forestland to the river shows upstream farmers is 116.87 m and 223.21 m, respectively. Upstream farmers’ social network is weaker than midstream farmers, which is not significant.

### 3.2. Choice Experiment Estimation

#### 3.2.1. RPL Model

As shown in Table 4, Model 1 and Model 2, respectively, estimate the preference of upstream and midstream farmers to participate in ecological compensation programs with RPL. For each respondent, three choice experiments were performed, and each choice had three options; we eventually obtained 2610 (290 × 3 × 3) groups of observations. In the RPL, agricultural water quality and compensation years are random parameters, and livestock and poultry breeding, agricultural waste recovery rate, compensation amount, and ASC are fixed parameters. To analyze the individual heterogeneity of farmers, we present the interaction term between ASC and farmers’ characteristic variables in Model 3 and Model 4. The adjusted R^2^ of Model 3 and Model 4 are higher than those of Model 1 and Model 2. This indicates that the overall goodness-of-fit of the models is improved after adding the interaction terms. Therefore, the following mainly describes Model 3 and Model 4. The ASC coefficient is negative in Model 1, which means that the upstream farmers perceived that they would derive utility from improvements in the policy. However, the ASC coefficient is positive in Model 2, which means the midstream farmers prefer to maintain the status quo. In Model 3 and 4, the ASC coefficient is not significant but positive, indicating that it does not reject the scheme without governance measures.

The estimated coefficients for the “compensation year”, “livestock and poultry breeding” and “cash requirement” variables are significant, and the influence direction is the same between upstream and midstream farmers. These results are expected, as farmers would be more willing to participate a program that offers them with an improvement in the key outcomes. The more interesting findings are the different significances and importance of “agricultural water quality” and “agricultural waste recycling rate” variables, which indicate upstream and midstream farmers’ heterogeneous preferences to their participation in this PES programs. The results show that the estimated coefficient of agricultural water quality is negative and significant at 5% level for midstream, but not significant for upstream. The Xin’an River water quality monitoring station is located in the middle stream, and the local government will conduct the testing once a month, which seriously affects the livelihood of local farmers, so that the farmers’ desire to reduce water quality shows more eagerly. The coefficient of agricultural waste from upstream samples is negatively significant at the 10% level, indicating that upstream farmers expect a lower recycling rate of agricultural waste, which is not significant for midstream farmers. The difference in initial conditions related to program administration might explain this result: the payments for the recycling agricultural waste to midstream farmers are higher than those to upstream farmers. Hence, the upstream farmers prefer to handle with a low rate of recycling rate of agricultural waste to save costs in planting and other agricultural works.

When ASC is interacted with farm household characteristic variables, we find that gender, age, education, number of laborers, forestland area, and distance from forestland to river differed between upstream and midstream farmers. For upstream farmers, the estimated coefficients of age and forest area are negatively significant, while the coefficient of distance from forest to river was positively significant. It shows that the farmers with older age, larger forest area and closer to the Xin’an River are willing to accept more compensation in the policy, as expected. For midstream farmers, the estimated coefficients of gender and education are positively significant, indicating that there is a significant difference between male and female farmers ‘participation of ecological compensation in the midstream. Additionally, the lower the education level, the higher the willingness of farmers to accept compensation in ecological compensation programs, which may be related to farmers’ perceptions of ecology. It is interesting that the estimated coefficient of the number of labor force is negatively significant for midstream farmers. It may indicate that the ecological compensation programs prohibit fishing resulting in the loss of employment opportunities for farmers, but the compensation payment cannot compensate for the loss of income. Therefore, the higher the number of laborers, the more serious the loss of farmers and the higher the willingness to be compensated. The estimated coefficients of forestland slope and the distance from the residence to the river are not significant both in the upstream and midstream. These results indicate that these two variables did not significantly affect the willingness of farmers to participate in ecological compensation. Overall, the results indicate that the natural capital and human capital has a greater influence on upstream farmers’ WTA, while the human capital has a greater influence on on midstream farmers’ WTA.

#### 3.2.2. WTA Estimations

According to Formula (5), we represent the willingness to be paid by upstream and midstream farmers for each attribute (Table 5). To protect agricultural water quality, midstream farmers expect to receive a subsidy of RMB 24.94 per month, but upstream farmers’ willingness to be paid for agricultural water quality is 0. Both upstream and midstream farmers have negative willingness to be compensated for the compensation years. This means that the amount of compensation does not increase, and farmers also expect to extend the compensation years to increase the farmers’ income [71]. In terms of livestock and poultry breeding, the amount of compensation expected by upstream farmers every month is RMB 55.36, which is about 1.5 times that of the midstream. Among all attributes, the largest difference between upstream and midstream is the willingness to be paid for the recycling rate of agricultural waste. The willingness of upstream farmers to recycle agricultural waste is high per month, while the midstream is 0 RMB/month.

Judging from the overall compensation amount, the upstream (149.88 RMB/month) is significantly higher than the midstream (57.40 RMB/month). This may be related to current compensation. In order to protect the ecological environment of Xin’an River, the upstream sacrificed its economic development, but the local farmers received very little cash compensation and only received organic fertilizer compensation. The midstream farmers received compensation not only for the reservoir area but also for the fishing boats and fishing gear of the returning fishermen. Another important finding is that when considering the farmers’ heterogeneity, the WTA is higher. It illustrates that farmers’ heterogeneity should be considered in policy making [72].

### 3.3. ABM Estimation

In the CE model, we found that human capital and natural capital influence farmers’ participation in PES. Nonetheless, social capital is also an important factor that influences farmers’ choice with their social network and social trust. In a traditional econometric model, this interaction could not be observed easily, especially the long-term interactions.

**Figure 4:**
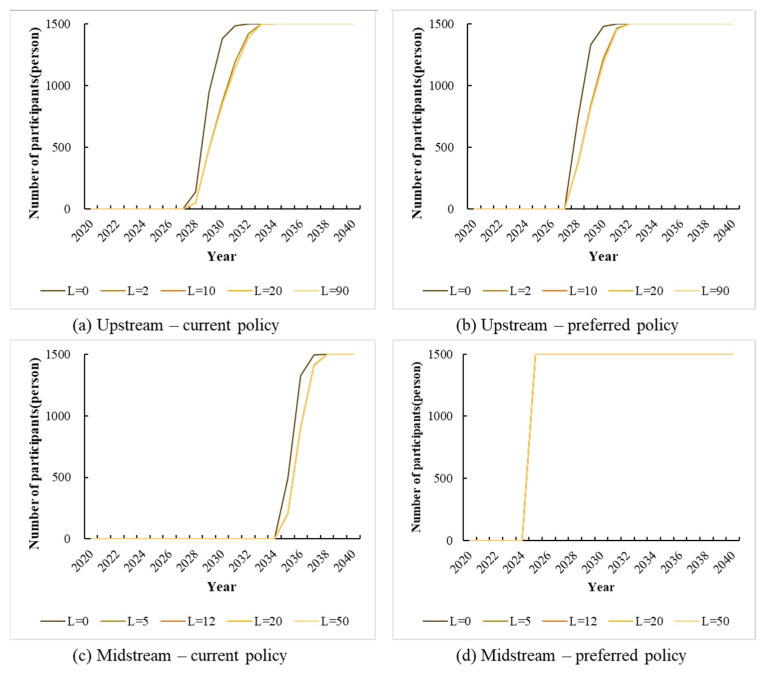
Impacts of different social network sizes in different scenarios. (**a**) the changes in the willingness of upstream farmers to participate in the current policy scenario; (**b**) the changes in the willingness of upstream farmers to participate in the policy preference scenario; (**c**) the changes of the willingness of the midstream farmers to participate in the current policy scenario; (**d**) the changes in the willingness of upstream farmers to participate in the policy preference scenario.

Hence, we established an agent-based model to simulate farmers’ social trust and social networks in Xiuning and She counties under two different policy scenarios with Net logo software in four models, respectively (see details in Supplementary Materials). In each agent-based model, 1500 farmers were initialized and simulated for 20 years. To avoid the randomness of a single experiment affecting the reliability of the results, we conducted 30 parallel experiments. The final results were obtained by averaging the 30 experiments’ outcomes for each model (Figure 3 and Figure 4).

Firstly, we conducted a comparison with the changed social trust parameters in the model. The results show that the responses of upstream and midstream farmers to the ecological compensation policy are different: 3.5% of farmers in the upstream are willing to participate in the policy in 2028; 14.0% of farmers in the midstream are willing to participate in the ecological compensation policy in 2035. This indicates that, in the current policy, both the upstream and midstream farmers are reluctant to participate the programs. However, under the preferred compensation policy, farmers’ performances are much better; for example, in 2028 and 2025, the upstream and midstream farmers wish to participate in these programs, respectively. We also found that under the preference policy, the midstream farmers achieved the goal of full participation faster, while under the current policy, farmers in the upstream reaches their goals faster. It may be due to the social trust iteration that farmers’ preferences have changed. In 2020–2024, the number of participants in the upper and middle reaches of farmers in both scenarios is 0. This implies a period during which farmers’ preferences change.

This paper also considers the impact of social network differences on farmers’ willingness to participate. The number of farmers with mutual labor relations (L) in the research data is used as an indicator of social network variables. The values of the parameters in the model depend on the minimum, quartiles, and maximum values of the data. The results are shown in Figure 4.

Overall, social networks have a significant effect on farmers’ willingness to participate, especially under the status quo policy scenario. Figure 4a–c reflect the trend that the larger the size of social networks, the lower the willingness of farmers to participate. The process of decision making is involved when the farmer chooses between whether to participate in two options. According to the limited rationality paradigm [73] described, in this case, farmers make judgments with the limited information they receive from their neighbors. Farmers with larger social networks have access to more information. Positive versus negative information makes it difficult for farmers to make decisions. At the same time, the opportunity cost of farmers’ participation in ecological compensation is difficult to be fully compensated. According to prospect theory [74], we know that people are more sensitive to losses than gains. Therefore, farmers choose not to participate to avoid losses. In the preferred policy scenario, farmers’ willingness to participate is significantly higher than in the current policy scenario. In Figure 4b, the difference between L = 0 and L = 90 is small; in Figure 4d, there is no difference at all. It could be that the implementation of these policies is beneficial to the farming households, and rational farming households will spontaneously participate in the policies in the presence of the identified benefits.

We can also see that social networks have a greater impact on upstream farmers under the current programs. It indicates that the midstream farmers are stable to participate in the preference programs with social network affection. Nevertheless, in the other three scenarios, the tendency is not distinct. This significance includes that the social network of upstream farmers’ negatively influences their participation in the current programs. The heterogeneity of the social network should be noted.

## 4. Discussions, Conclusions and Policy Implications

### 4.1. Discussions

In this paper, the discussion of PES is largely about ecological compensation in the basin, and the research conclusions provide policy guidance for similar areas such as the Xin’an River basin. However, in practice, different geographic and socio-economic areas may have different ecological compensation programs. In mountainous areas of developing countries, the larger the social network, the more likely to achieve the negative information because the definition of this variable (social network) is to communicate with his farmer friends [75]. If these farmers have negative emotions, they will influence each other. Nevertheless, different compensation projects have varying impacts in different regions, as well as different final external benefits. In addition, there are differences in costs and benefits associated with various compensation plans. Hence, future studies need to further refine the corresponding relationship between compensation projects and ecological indicators, measure their external benefits separately and calculate compensation standards with more application value based on adoption costs and private benefits.

Furthermore, this paper provides a feasible way of quantifying the external benefits of the river basin ecological compensation project, but the improvement of the river basin ecological environment also improves the welfare level of the residents outside the region (by improving water quality, for instance), and this benefit is not included in this paper. Based on existing research, it appears that spatial characteristics of willingness to participate in the PES programs have been confirmed. In a previous study, for example, the willingness to pay of residents in Ansai, Xi’an and Beijing were quantified to protect and restore the Loess Plateau, and Beijing residents’ willingness to pay was more than twice that of those in Ansai and Xi’an [76]; researchers have also determined that residents in the Guadalquivir River basin are willing to pay not just to improve the water quality in their area, but to improve other areas as well [77]. The water quality of the segment has a willingness to pay. Therefore, follow-up research needs to further explore the welfare changes of residents outside the region, so that the quantitative results of external benefits used in ecological compensation projects are more comprehensive and accurate.

### 4.2. Conclusions

We used a CE method and ABM in Xin’an River basin, China, to evaluate the farmers’ preferences and estimate the willingness-to-accept measures for a hypothetical payment for PES programs. These models allow us to evaluate the relative importance of changes in program attributes and farmers’ characteristics. Determining farmers’ preferences and WTA for PES programs is a vital process in improving the current ones in the long term.

Our results suggest that farmers both upstream and midstream have preferences for compensation years, cash requirements and livestock farming. It was also found that farmers resumed logging behavior when monetary payments to farmers participating in PES were stopped [78]. In this way, it seems understandable that farmers in the Xin’an River basin expect to extend their compensation years. Of the 290 farmers we surveyed, 130 were in the upper reaches and 160 in the lower reaches. There is also spatial heterogeneity in their preferences. Upstream farmers expect lower agricultural waste recycling rates, and midstream farmers expect lower agricultural water quality. Age, forest area, and distance from forest to river affect upstream farmers’ choice of policy; gender, education, and number of laborers affect midstream farmers’ choice. It implies that the human capital influences the midstream farmers’ participation, while the human capital and natural capital influence the upstream farmers’ participation.

We coded the CE results into the ABM to simulate farmers’ willingness to participate in a “bottom-up” policy scenario that takes into account farmers’ heterogeneity and social capitals, which are not easily captured in the CE model. The results show that farmer willingness to participate increases significantly when farmer preferences are incorporated into the policy design, and the results of this study confirm the findings of other studies that non-economic factors, such as participation in program design, are key determinants of sustained participation in PES programs [79,80,81]. The results also indicate that social trust has less influence than social networks in both samples. Moreover, farmers from upstream are more influenced than farmers from midstream by their social network. We discussed this finding with two reasons. First, in social trust, there is a game strategy, which means people will not change their choices in case someone cheats. Cooperation and deception will always be at play, so the outcome of the final strategy does not change. Moreover, the education level of the local farmers is not high (Table 3), so the strategies may be economically rational. Second, in farmers’ social networks, there is a strong tie, which is not necessarily positive. This evaluation may be combined with this negative decision making. This is named learning affection. The higher the number of nodes in the network, the more complex and hesitant this effect becomes. People with high social trust believe in the wrong strategy if everyone else is cheating, which causes homogeneity and echoing effects. This also illustrates why the influences of strong ties in social network are greater than those of social trust.

### 4.3. Policy Implications

Our results highlight the need to take local farmers’ heterogeneity into consideration in the design and promotion of PES programs. Locally estimated WTA values are vital in quantifying the benefits of PES programs. We point out three recommendations from the results. First, the design of ecological compensation policy should be optimized by considering the heterogeneity of local farmers. Farmers are the implementers of the policy, and only by fully understanding their needs can we maximize their willingness to participate. Second, the policy should be targeted. Upstream and midstream farmers’ preferences are spatially heterogeneous, which means that there is no one-size-fits-all policy. The upstream government should raise the price of agricultural waste recycling, while the midstream government should moderately relax the restrictions on farmers’ behavior while ensuring water quality. For example, the government can cooperate with local enterprises to build facilities to treat livestock and poultry manure so that farmers can carry out farming. Finally, the government should increase the amount of compensation to farmers and the length of compensation years. Farmers will be less motivated when their losses are not equal to their gains.

## Figures and Tables

**Table 1:** Description of attributes and levels.

Attributes	Levels
Livestock and poultry breeding	total prohibition = 1; rationalization = 0
Agricultural water quality	grade I = 1; grade II = 0
Agricultural waste recycling rate	75%; 80%; 85%; 90%
Compensation years	3 years; 5 years; 7 years; 9 years
Cash requirement	25 RMB/month; 50 RMB/month; 75 RMB/month

**Table 2:** A sample choice set.

Attributes	Alternative A	Alternative B	Alternative C
Livestock and poultry breeding	total prohibition 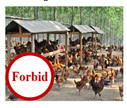	total prohibition 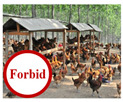	Neither alternative A, nor B. I would maintain current farm management
Agricultural water quality	water quality grade II 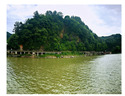	water quality grade I 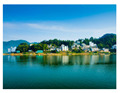	
Agricultural waste recycling rate	80% 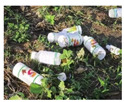	75% 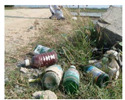	
Compensation years	7 years 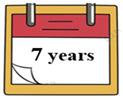	7 years 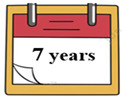	
Cash requirement /month	75 yuan/month 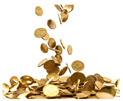	50 yuan/month 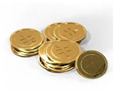	

**Table 3:** Descriptive statistics.

Variables	Description	Total	Upstream	Midstream	Difference in Means
		Mean (SD)	Mean (SD)	Mean (SD)	
Gender	Gender				0.04
	(Male = 1; Female = 0)	0.62	1.40	1.36	
		(0.49)	(0.49)	(0.48)	
Age	Age	61.33 (10.67)	61.38 (12.09)	61.29 (9.33)	0.08 ***
Education	Education years(year)	6.91 (3.62)	5.48 (3.96)	8.08 (2.80)	−2.60 ***
Number of laborers	Number of household labor force	2.94 (1.41)	3.30 (1.51)	2.64 (1.23)	0.66 **
Forest land area	Forest land area (ha)	1.02 (2.05)	1.97 (2.77)	0.24 (0.30)	1.72 ***
Forest land slope	Slope of forest land (Gentle = 1; Generally steep = 2; Steep = 3)	1.94 (0.60)	1.99 (0.62)	1.90 (0.57)	0.09
Distance from residence to river	Distance from the living house to Xin’an River(m)	248.48 (239.50)	116.87 (127.30)	355.41 (254.94)	−238.54 ***
Distance from forest land to river	Distance from the forest land to the Xin’an River(m)	304.48 (447.81)	223.21 (356.12)	370.35 (499.73)	147.14 ***
Social network	Numbers of farmers with mutual working relations	15.78 (31.65)	14.25 (16.03)	17.02 (40.11)	−2.77

Note: T tests test for differences in means of characteristics between upstream and middle stream. ***, ** indicate significance at 1%, 5%level.

**Table 4:** RPL estimations.

		RPL Model		RPL Model with Interaction	
		Model 1	Model 2	Model 3	Model 4
		Upstream	Midstream	Upstream	Midstream
Variables		Coefficient (Str. Error)	Coefficient (Str. Error)	Coefficient (Str. Error)	Coefficient (Str. Error)
ASC		−6.609 ** (2.670)	1.752 *** (0.466)	0.288 (2.005)	1.208 (1.775)
Agricultural water quality	Mean	−1.956 (1.250)	−0.838 *** (0.305)	−0.636 (0.496)	−1.571 ** (0.670)
	Standard deviation	6.093 * (3.139)	0.735 (1.029)	1.691 * (0.997)	3.138 * (1.722)
Compensation years	Mean	0.530 ** (0.262)	0.148 ** (0.065)	0.391 *** (0.109)	0.168 *** (0.061)
	Standard deviation	1.662 (1.042)	0.612 ** (0.308)	0.834 *** (0.256)	0.263 (0.288)
Livestock and poultry breeding		−6.447 ** (2.572)	−1.572 *** (0.489)	−4.484 *** (1.143)	−2.213 *** (0.748)
Agricultural waste recycling rate		−13.065 (8.614)	−7.592 (4.642)	−8.047 * (4.394)	−5.513 (4.488)
Cash requirement		0.112 ** (0.044)	0.059 *** (0.013)	0.081 *** (0.020)	0.063 *** (0.014)
ASC × Gender				−0.469 (0.598)	1.119 *** (0.427)
ASC × Age				−0.057 ** (0.024)	0.024 (0.022)
ASC × Education				−0.064 (0.074)	0.142 * (0.078)
ASC × Number of laborers				−0.099 (0.171)	−0.348 ** (0.157)
ASC × Forestland area				−0.848 *** (0.262)	0.033 (0.582)
ASC × Forestland slope				−0.126 (0.418)	−0.327 (0.332)
ASC × Distance from residence to river				−0.001 (0.002)	−0.0002 (0.001)
ASC × Distance from forestland to river				0.003 *** (0.001)	0.466 (0.0004)
Obs		1170	1440	1170	1440
Log likelihood		−352.514	−471.811	−305.029	−460.116
AIC		723.0	961.6	660.1	970.2
R^2^		0.175	0.105	0.286	0.127

Note: ***, **, * indicate significance at 1%, 5%, 10% level.

**Table 5:** WTA estimates and 95% confidence interval for each attribute.

	RPL Model		RPL Model with Interaction	
	Model 1	Model 2	Model 3	Model 4
Attributes	Upstream	Midstream	Upstream	Midstream
Agricultural water quality	(0) (−2.5, 176.2)	14.20 (2.8, 43.5)	(0) (−2.8, 37.4)	24.94 (2.8, 80.1)
Compensation years	−4.73 (−5.3, −0.6)	−2.51 (−3.2, −0.6)	−4.83 (−5.1, 4.1)	−2.67 (−2.8, −1.4)
Livestock and poultry breeding	57.56 (7.1, 459.6)	26.64 (7.2, 76.7)	55.36 (18.8, 156.5)	35.13 (8.3, 102.2)
Agricultural waste recycling rate	(0) (−19.2, 1198.0)	(0) (−17.8,505.8)	99.35 (−4.8, 387.4)	(0) (−36.4, 397.5)
Total	52.83 (−19.9, 1833.1)	38.33 (−10.9, 625.3)	149.88 (6.1, 577.3)	57.40 (−28.1, 578.4)

Note: WTA measures associated with attribute coefficients that are not significant at the 0.1 level are designated with square brackets and set to zero.

## Data Availability

No new data were created or analyzed in this study. Data sharing is not applicable to this article.

## Supplementary Materials

The following supporting information can be downloaded at: https://www.mdpi.com/article/10.3390/ijerph19127190/s1, Text S1: Independence of Irrelevant Alternatives (IIA) test; Text S2: ODD description of agent-based model; Table S1: Upstream-Remove Alternative A; Table S2: Midstream-Remove Alternative A; Table S3: Detailed description of state variables; Table S4: Probability of farmer’s choice of improved versus status quo scenario; Table S5: Upstream and midstream social network data parameter values; Figure S1: Impact of social trust on farmers’ choices. [49,59,60,68,70,73,82].
