# Supplementary material for: Considering Farmers’ Heterogeneity to Payment Ecosystem Services Participation: A Choice Experiment and Agent-Based Model Analysis in Xin’an River Basin, China
Source: Int J Environ Res Public Health. 2022 Jun 11;19(12):7190. doi: 10.3390/ijerph19127190 (PMC9222710; doi:10.3390/ijerph19127190)
Supplement: Supplementary file 1 [file ijerph-19-07190-s001.zip › ijerph-1742049-supplementary.pdf]

Supplementary Materials

Considering Farmers' Heterogeneity to Payment Ecosystem Services Participation: A Choice Experiment and Agent-Based Model Analysis in Xin'an River Basin, China

Shengnan Li [1], Baohang Hui [1], Cai Jin [1], Xuehan Liu [1], Fan Xu [2], Chong Su [3] and Tan Li [1,*]

[1] College of Economics and Management, Anhui Agricultural University, Hefei 230036, China; lishengnan19970406@163.com (S.L.); hbh@stu.ahau.edu.cn (B.H.); JinC612@outlook.com (C.J.); L18053796938@163.com (X.L.)
[2] College of Economics and Management, South China Agricultural University, Guangzhou 510642, China; xf0312@stu.scau.edu.cn
[3] Institute of Agriculture Remote Sensing and Information Technology, College of Environmental and Resource Sciences, Zhejiang University, Hangzhou 310058, China; suchong@zju.edu.cn
* Correspondence: litan@ahau.edu.cn

Supplementary Text S1: Independence of Irrelevant Alternatives (IIA) test

We conducted the Independence of Irrelevant Alternatives (IIA) test to select the appropriate model for the assessment of farmers' preferences. Under the IIA hypothesis, the magnitude of the choice probability does not change due to an increase or decrease in the choice alternatives. This is a strict assumption of Multinomial logit (MNL). If the IIA test is passed, then the MNL model can be used. If not, then the Random parameter logit (RPL) model can be used.

We performed the IIA test separately for upstream and midstream farmers. When alternative A is removed, the IIA hypothesis is rejected. When alternative B and alternative C are removed, the IIA test is passed. However, having one hypothesis rejected indicates that the three alternatives are not independent of each other. Here, the full model incorporating the three alternatives is denoted as "All". If alternative A is removed, it is denoted as "N-A". The results are shown in Table S1 and Table S2.

Supplementary Text S2: ODD description of agent-based model

The model description follows the ODD (Overview, Design concepts, Details) protocol for describing individual- and agent-based models[1], as updated by Grimm et al. [2].

S2.1. Purpose

The purpose of this model is to simulate how differences in policy preferences and social capital of farmers in the upper and middle reaches of the Xin'an River affect farmers' willingness to participate in ecological compensation policies.

S2.2. Agents, state variables and scales

The social agent of the model is the farmer. The state variables of farmers include gender, age, education, number of household laborers, area of forest land, slope of forest land, distance from residence to river, and distance from forest land to river. Among the above variables, only age was time-varying. These variables were obtained from the field survey of the study participants. We obtained the values of the state variables based on the research data. To reflect the heterogeneity of farmers in the model, we set random numbers between the minimum and maximum values of the state variables to assign values to farmers (Table S3). The simulation starts at 2020 when the household survey data was collected. Each time step represents 1 year. The model is run for a total of 30 years. The model can simulate the participation of farmers in the upstream and midstream of Xin'an River in the ecological compensation policy during 2020-2040. We set up 1,500 farmers in the upstream and midstream respectively.

S2.3. Process overview and scheduling

Figure 3 shows the flow chart of the structure of ABM. The modeling process can be divided into three parts: initialization, alternative parameter values for simulation, and output results. The initialization phase focuses on giving each farmer an initial willingness value to participate in the ecological compensation policy based on the questionnaire research data. In the simulation stage, the following operations are performed in each time step. (1) Willingness value increase or decrease: Farmers increase or decrease the initial willingness value according to the results of the choice experiment; (2) Social interaction: Farmers interact with people around them.

S2.4. Design concepts

Theoretical and empirical background. The choice experiment modeling framework relies on the characteristics theory of value[3] and the random utility theory[4]. Lancaster (1966) assumes that a good may be defined by a set of characteristics. Therefore, the value of a commodity is the sum of all its characteristic values. In the selection experiment this theory can be expressed as describing the commodity in terms of a set of attributes. The theory of stochastic utility proposed by McFadden (1973) holds that the true utility of consumer consumption of goods is divided into observable definite utility and non-observable random utility. The ecological compensation policy adopted the "top-down" institution for environmental regulation through economic incentives. Based on empirical understanding, the extent to which farmers' willingness to participate in the policy may vary. Recognizing this heterogeneity prompted us to design an experiment from farmer needs, making it possible to study each farmer's preferences for policy options. Thus, following the empirical knowledge derived from the choice experiment analysis, the model simulated the preferences of the current and designed schemes by each individual farmer. Decision-making processes were involved when selecting between the two schemes[5]. Theoretical background is also depicted by the paradigm of bounded rationality well situated within the theory of Satisficing[6], in which case each farmer would make a decision depending on limited information obtained from neighbors. For instance, farmers' preference can be influenced by the behavior of neighbors only within a certain range.

Individual decision-making. A farmer's preference for policy, along with individual characteristics and social interactions, affects the farmer's participation in one of the policy schemes. An individual farmer aims to maximize the benefits or minimize risks of choosing a policy scheme by valuing cost opportunities between different choices. For instance, when a farmer maintains the participating status in the current policy scheme, the farmer estimated that the alternative would not be able to cover the costs of switching. The decision-making process was simulated through the estimation of the probability of staying the current policy scheme comparing to switching to the other.

Emergence. The participation rate of ecological compensation policies depends on the farmer's decision, which in turn depends on his preference for the policy, individual characteristics, and social interactions.

Interaction. The interaction between farmers is direct and local. They exchange information by identifying each other. Their behavior is influenced by the willingness of farmers who have mutual labor relations.

Sensing. Farmers' agents can perceive their own properties and the slope of the forest land, its area, and the distance to the river. They can also perceive the willingness of their neighbors to participate in the policy. These perceptions will influence the farmer's participation decision.

Stochasticity. The gender, age, education level, forestland area, forestland slope, distance from residence to river, and distance from forestland to river of the farmers in the model were randomly generated based on the minimum and maximum values between the corresponding variables obtained in the research.

Heterogeneity. ABM focuses on the micro-behavior of human subjects[7], such as individual characteristics of farmers (gender, age, education, etc.) and the degree of preference for policies are characterized by heterogeneity.

S2.5. Details

Initialization & input data. The model uses data from the results of the choice experiment and field research data. In the initialization stage, we assign values to the initial willingness and state variables of the farmers separately. The initial willingness of all farmers is 0. The state variables of farmers are randomly generated between the minimum and maximum values of the corresponding variables from the research data. The model is experimental and hence does not use real-world data as input data to represent time-varying processes.

Submodules. There are two submodules in the model, with details as follows.

(1) Changes in farmers' willingness to participate.

We used stata15.0 software to predict the probability of upstream and midstream farmers' choices of alternatives. Taking the upstream as an example, we surveyed 130 farmers and each farmer made 3 choices, so we obtained 130 × 3 = 390 choices. We used stata15.0 software to obtain the probability of each farmer choosing alternative A, alternative B, and alternative C. Among them, both alternative A and alternative B are preference alternatives and alternative C is the status quo one. We calculated that the sum of the probabilities of upstream farmers choosing Alternative A and Alternative B among 390 choices is 203.19, so the average probability of farmers choosing the preferred alternative is 203.19/390 = 0.52. The sum of the probabilities of upstream farmers choosing Alternative C among 390 choices is 186.81, so the probability of farmers choosing the status quo alternative is 186.81/390 = 0.48. The midstream probability values were also calculated in this way, and the results are shown in Table S4.

The capital endowment of the farmer also affects his choice of scenario, such as gender, age, education level, number of laborers, distance from residence to the river, distance from forest to the river, forest area, and slope of the forest have different effects on the willingness of the farmer to participate. According to the results of RPL in Table 4, we can see that in the upstream, the age of farmers, the area of forest land and the distance from the forest land to the river affect the farmers' choice of program. While in the downstream, gender, education and the number of household labor will farmers' choice.

(2) Social interaction.

According to the limited rationality paradigm[6] described, each farmer will make decisions based on the limited information received from neighbors. Social capital affects farmers' decisions. Therefore, we considered the influence of social trust and social networks on farmers' choices.

• Social trust: We set the parameter values of social trust (Trust-rate) to 0, 0.25, 0.5, 0.75, and 1, respectively, and conducted 30 parallel tests for each model. The obtained results are shown in Figure S1. We found that the degree of social trust did not reflect significant differences in the models.

• Social networks: To analyze the influence of social network size on farmers' choice, we chose "the number of farmers with mutual labor relations" as the indicator in the data obtained. In the ABM, the parameter is taken as the quartile of the "number of farmers with mutual labor relations" in the upper and middle streams (Table S5). When the number of willing participants around the farmer exceeds the size of the farmer's social network, the farmer will be willing to participate in the policy.

Table S1. Upstream-Remove Alternative A.

Variables	N-A	All	Difference	S.E.
Gender	-0.678	-0.401	-0.277	0.099
Age	-0.003	0.017	-0.020	0.003
Education	0.052	0.013	0.039	0.012
Number of household labor	-0.139	-0.024	-0.115	.
forestland area	0.007	0.061	-0.053	.
forestland slope	-0.021	0.025	-0.046	0.023
Distance from residence to river	0.001	-0.001	0.001	0.000
Distance from forestland to river	0.001	0.000	0.001	0.000
_cons	0.201	-1.005	1.206	0.250

Test: Ho: difference in coefficients not systematic; chi2 = 16.22; Prob>chi2 = 0.0625.

Table S2. Midstream-Remove Alternative A.

Variables	N-A	All	Difference	S.E.
Gender	0.619	0.118	0.500	0.105
Age	-0.009	0.017	-0.026	0.008
Education	-0.069	0.006	-0.075	0.019
Number of household labor	0.178	0.079	0.099	0.053
forestland area	-0.004	0.036	-0.041	.
forestland slope	0.086	0.097	-0.011	0.059
Distance from residence to river	0.000	-0.001	0.000	0.000
Distance from forestland to river	0.000	0.000	0.000	0.000
_cons	-0.076	-2.083	2.007	0.612

Test: Ho: difference in coefficients not systematic; chi2 = 43.85; Prob>chi2 = 0.0000.

Table S3. Detailed description of state variables.

	Upstream		Midstream	
Variables	Min.	Max.	Min.	Max.
Gender	0	1	0	1
Age	25	89	34	83
Education/years	0	15	0	16
Number of household labor/person	1	10	0	8
forestland area/ha	0	21	0	3
forestland slope	0	3	1	3
Distance from residence to river/m	0	800	10	1000
Distance from forestland to river/m	0	1500	0	3000

Table S4. Probability of farmer's choice of improved versus status quo scenario.

	Upstream	Midstream
Alternative A	27%	44%
Alternative B	25%	33%
Alternative C	48%	23%

Table S5. Upstream and midstream social network data parameter values.

	Min.	25%	50%	75%	Max.
Upstream	0	2	10	20	90
Midstream	0	5	12	20	50

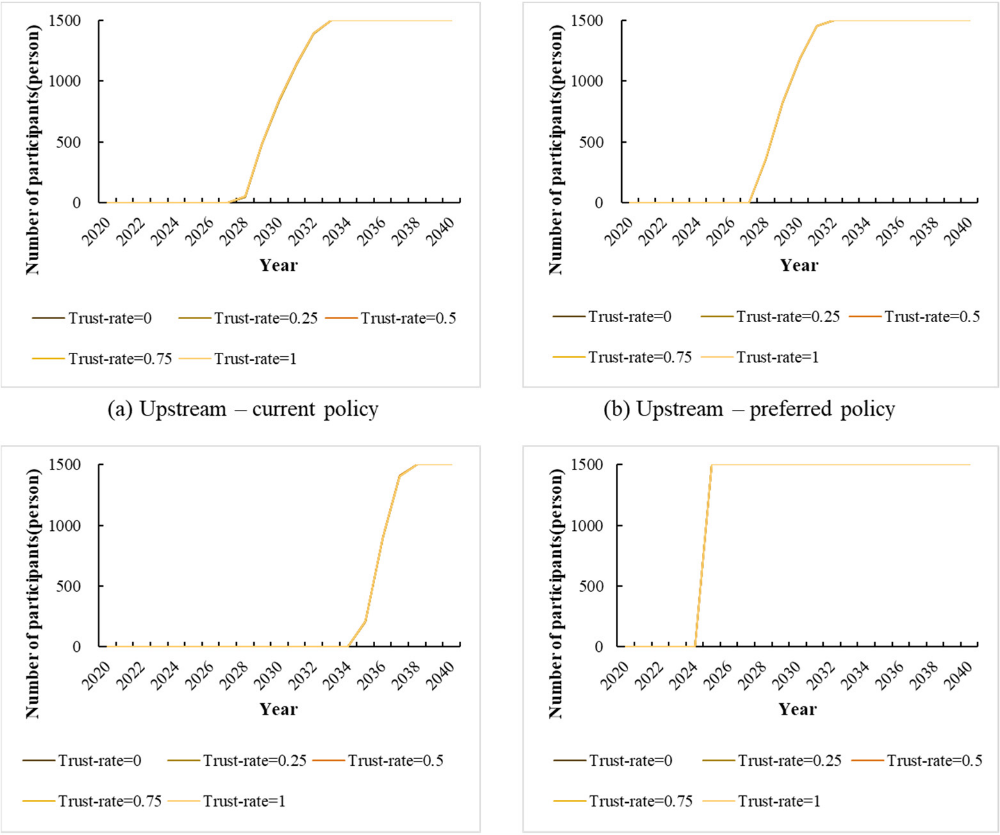

(a) Upstream – current policy (b) Upstream – preferred policy

(c) Midstream – current policy (d) Midstream – preferred policy

Figure S1. Impact of social trust on farmers' choices.

References

1. Grimm, V.; Berger, U.; Bastiansen, F.; Eliassen, S.; Ginot, V.; Giske, J.; Goss-Custard, J.; Grand, T.; Heinz, S.K.; Huse, G.; et al. A standard protocol for describing individual-based and agent-based models. *Ecol. Model.* **2006**, *198*, 115–126. https://doi.org/10.1016/j.ecolmodel.2006.04.023.
2. Grimm, V.; Railsback, S.F.; Vincenot, C.E.; Berger, U.; Gallagher, C.; DeAngelis, D.L.; Edmonds, B.; Ge, J.; Giske, J.; Groeneveld, J.; et al. The ODD Protocol for Describing Agent-Based and Other Simulation Models: A Second Update to Improve Clarity, Replication and Structural Realism. *J. Artif. Soc. Soc. Simul.* **2020**, *23*, 7. http://dx.doi.org/10.18564/jasss.4259.
3. Lancaster, K.J. A New Approach to Consumer Theory. *J. Political Econ.* **1966**, *74*, 132–157.
4. McFadden, D. Conditional logit analysis of qualitative choice behavior. In Frontiers of Econometrics; Zarembka, P.E., Ed.; Academic Press: New York, NY, USA, 1973.
5. Groeneveld, J.; Mueller, B.; Buchmann, C. M.; Dressler, G.; Guo, C.; Hase, N.; Hoffmann, F.; John, F.; Klassert, C.; Lauf, T., Theoretical foundations of human decision-making in agent-based land use models—A review. *Environ. Model. Softw.* **2017**, *87*, 39-48.
6. Simon, H.A. Rational choice and the structure of the environment. *Psychol. Rev.* **1956**, *63*, 129–138. http://dx.doi.org/10.1037/h0042769.
7. Wang, Y.; Zhang, Q.; Li, Q.; Wang, J.; Sannigrahi, S.; Bilsborrow, R.; Bellingrath-Kimura, S.D.; Li, J.; Song, C. Role of social networks in building household livelihood resilience under payments for ecosystem services programs in a poor rural community in China. J. Rural Stud. **2021**, *86*, 208–225. http://dx.doi.org/10.1016/j.jrurstud.2021.05.017.
